# Chronic widespread pain and its associations with quality of life and function at a 20- year follow-up of individuals with chronic knee pain at inclusion

**DOI:** 10.1186/s12891-019-2976-3

**Published:** 2019-12-09

**Authors:** Stefan Bergman, Carina Thorstensson, Maria L. E. Andersson

**Affiliations:** 1 Primary Health Care Unit, Department of Public Health and Community Medicine, Institute of Medicine, The Sahlgrenska Academy, University of Gothenburg, Gothenburg, Sweden; 2 Department of Clinical Sciences, Rheumatology, Lund University, Lund, Sweden; 3 Spenshult Research and Development Center, Bäckagårdsvägen 47, SE-302 74 Halmstad, Sweden; 4 Department of Clinical Neuroscience and RehabilitationThe Sahlgrenska Academy, Institute of Neuroscience and Physiology, University of Gothenburg, Gothenburg, Sweden

**Keywords:** Knee osteoarthritis, Chronic widespread pain, Patient reported outcomes

## Abstract

**Objective:**

To study the prevalence of chronic widespread pain (CWP) and chronic regional pain (CRP), and their association to quality of life, pain, physical function at a 20-year follow-up in a population based cohort with chronic knee pain at inclusion.

**Methods:**

121 individuals (45% women, mean age 64 years, range 54–73) with chronic knee pain from a population-based cohort study, answered a questionnaire and had radiographic knee examination at a 20-year follow-up. The responders were divided into three groups according to reported pain; individuals having no chronic pain (NCP), chronic widespread pain (CWP) and chronic regional pain (CRP). Pain and physical function were assessed using Knee injury and Osteoarthritis Outcome Score (KOOS). Health related quality of life (HRQL) was assessed with Euroqol-5D-3 L (EQ5D) and Short form 36 (SF36). The associations between pain groups and KOOS, EQ5D, and SF36 were analysed by multiple logistic regression, controlled for age, gender and radiographic changes indicating knee osteoarthritis (OA).

**Results:**

The prevalence of CWP was 30%, and CWP was associated to worse scores in all KOOS subscales, controlled for age, gender and radiographic changes. CWP was also associated to worse scores in EQ-5D and in seven of the SF-36 subgroups, controlled for age, gender and radiographic changes.

**Conclusion:**

One third of individuals with chronic knee pain met the criteria for CWP. CWP was associated with patient reported pain, function and HRQL. This suggest that it is important to assess CWP in the evaluation of patients with chronic knee pain, with and without radiographic knee OA.

## Introduction

Pain is the most disabling symptom of osteoarthritis (OA), resulting in disability and inactivity, and a common reason to search medical care. Several studies have shown associations between OA and fibromyalgia, with a fibromyalgia prevalence of 5 to 10% in individuals with OA compared to 1 to 5% in the general population [1–5]. The overall prevalence of chronic widespread pain in the general population is estimated to 10% [6].

Although the association between radiographic knee OA and reported pain has shown to be weak [7], there is an association between presence of pain and synovitis, bone marrow oedema, and bone marrow lesions [8]. Associations have also been seen between radiographic severity, assessed with Kellgren & Lawrence score, and Western Ontario and McMaster Universities Osteoarthritis Index (WOMAC) pain score, especially with regard to OA severity in the patellofemoral compartment [9].

There is evidence for shared pain mechanisms in OA and fibromyalgia [10]. Pain in OA is thought to be associated to an increased excitability of both peripheral and central pain pathways, which in the end could cause sensitization and an increased risk of widespread pain [10–12]. Studies of pain trajectories in knee OA have identified a group of individuals with severe pain, not improving over time, which could represesent a group with a more chronic widespread pain [13, 14].

Chronic widespread pain (CWP) and fibromyalgia (FM) are common in other musculoskeletal diseases, as for example rheumatoid arthritis (RA), spondylarthritis (SpA), systemic lupus erythematosus (SLE) and polymyalgia rheumatica. CWP has substantial impact on measures of disease activity, function, and pain [1, 15–18].

In studies of knee OA, knee-related patient reported outcomes such as Knee injury and Osteoarthritis Outcome Score (KOOS) [19] and WOMAC [20], together with measures of quality of life, such as Short form 36 (SF36), are recommended [21]. These knee related outcomes are also used in clinical settings, to assess the patient’s perspective of their knee associated problems. KOOS and WOMAC are designed to measure local pain and physical discomfort from, for example, the knees, but also patients with knee OA and a concurrent fibromyalgia have been reported to have a worse score on WOMAC [2]. Other factors, that have been reported to influence KOOS, are age and gender [21, 22] . The knowledge of how other factors, for example CWP, influence these knee related scores recommended to use as core outcome in OA trials are lacking.

The objective was to study the prevalence of chronic widespread pain (CWP) and chronic regional pain (CRP), and their association to pain, physical function and quality of life as measured by KOOS, EQ5D and SF36, in a 20-year follow-up of a population-based cohort with chronic knee pain at inclusion.

## Method

### Participants

This cross-sectional study included 121 individuals that in 2010 participated in a 20-year follow-up of a longitudinal population-based cohort, that at baseline included 183 individuals with knee pain. In 2010 there were 156 individuals eligible for the 20-year follow-up [23] [23]. The 20-year follow-up included a questionnaire and a radiographic knee examination.

### Questionnaire

Pain was reported by a pain mannequin (a figure with 18 predefined body regions) [24]. Pain duration for at least three months was designated as chronic. Chronic widespread pain (CWP) was defined according to the ACR 1990 criteria for fibromyalgia [25], requiring pain in both sides of the body, in upper and lower body, and in the axial skeleton. Those with chronic pain, but not fulfilling criteria for CWP were considered as having chronic regional pain (CRP). Individuals with pain duration shorter than three months were categorised as having no chronic pain (NCP). The questionnaire included the Knee injury and Osteoarthritis Outcome Score (KOOS) consisting of 5 subscales, range 0–100 (best to worse); Pain (KOOS-Pain), other Symptoms (KOOS-Symptom), Function in daily living (KOOS-ADL), Function in sport and recreation (KOOS-Sport/Rec) and knee related Quality of life (KOOS-QOL) [19, 26]. Health related quality of life was assessed by the Euroqol-5D-3 L (EQ5D) questionnaire and the 36-item short form survey (SF36). The EQ5D questionnaire, range 0–1 (worse to best), includes five questions about mobility, self-care, usual activities pain/discomfort, anxiety/depression, each of which can take one of three responses [27]. The SF36 questionnaire, range 0–100 (worse to best), assess quality-of-life in eight health concepts: physical functioning (SF36-PF), role physical (SF36-RP), bodily pain (SF36-BP), general health (SF36-GH), vitality (SF36-VT), social functioning (SF36-SF), role emotional (SF36-RE), mental health (SF36-MH) [28, 29].

### Radiographic examination

The radiographs were obtained in a skyline view of patellofemoral (PF) joints, and posteroanterior radiographs of both TF joints were obtained in weight-bearing position using a fluoroscopy unit. The patients stood with almost their entire weight on the leg being examined, with the knee flexed 30–50°, and with the patella and the big toe touching the table of the fluoroscopy unit. Radiographic osteoarthritis (OA) was defined as joint space width (JSW) < 3 mm in the tibiofemoral compartment and/or JSW < 5 mm in the patellofemoral compartment. Tibiofemoral knee OA (TFOA) was defined as JSW < 3 mm in the tibiofemoral compartment and patellofemoral knee OA (PFOA) as JSW < 5 mm in the patellofemoral compartment [30, 31]. Osteophytes in both compartments were scored according to Ahlbäck [32]. Fifteen individuals were not having the radiographic examination, two had total knee replacement in both knees, and the other 13 were not able to get to the examination due to work or long travel distance.

## Statistics

Statistical analyses were performed using SPSS Statistics 21 software. All tests were two tailed and conducted at the 0.05 significance level. Chi-square test was used to test for differences in proportions between groups. Kruskal-Wallis with post hoc pairwise analysis was used for continuous variables when comparing more than two groups, and Mann-Whitney when comparing two groups, due to that some of the variables were not normally distributed. Correlations were performed by the Spearman’s test. Multiple logistic regression analyses were used to study the associations between pain groups and being in the worse half, according to median value, of KOOS, EQ-5D and SF36 at 20 years follow-up, respectively, controlled for age, gender and having or not having radiographic knee OA.

## Results

Thirty-five individuals rejected participation, with no significant difference in age, gender distribution or (body mass index) BMI compared to the participants. The participants were 45% women, mean age was 64 years with a range between 54 and 73 years and mean BMI was 27.9 kg/m2 with a range between 19.2 and 45.0 kg/m2, where 75% had BMI > 25 kg/m2.

### Prevalence of chronic widespread pain

Thirty percent of the included individuals reported CWP (*n* = 36), 48% (*n* = 58) CRP and, 22% (*n* = 27) NCP. Six percent reported that they had been diagnosed with fibromyalgia (FM). There was no significant difference in age or BMI between the pain groups, Table 1. There were more women in the CWP group compared to the CRP group. Forty-nine (41%) of those participating in the 20-year follow-up reported knee pain in at least one knee, and of those 7 (6% of all participants) reported knee pain only.

**Table 1:** Descriptives of the three pain groups, and between group differences at 20-year follow-up

	All Mean (95% CI)	NCP Mean (95% CI)	CRP Mean (95% CI)	CWP Mean (95% CI)	*P*-value
N (%)	121	28 (23)	57 (47)	36 (30)	
Age	64 (63–65)	63 (61–65)	64 (62–65)	63 (61–65)	0.904
Women %	45	54	33	56	0.061
BMI (kg/m^2^)	27.9 (27.1–28.7)	26.9 (25.2–28.4)	27.9 (26.9–28.9)	28.8 (26.8–30.8)	0.521
Painful regions (0–18)	3.8 (3.1–4.4)	0 (0–0)	2.9 (2.4–3.4)	8.1 (7.0–9.1)	< 0.001
Knee pain (%)	40	0	35	81	< 0.001
Knee OA (%)	51	44	52	55	0.707
TFOA (%)	17	12	14	26	
PFOA (%)	29	32	30	26	0.535^a^
TFPFOA (%)	5	0	8	3	
Osteophytes (%)	91	88	94	90	0.664
KOOS-pain (0–100)	72.8 (68.9–76.8)	84.8 (78.0–91.6)	74.2 (68.2–80.3)	61.6 (55.4–67.8)	< 0.001
KOOS-symptom (0–100)	74.3 (70.8–77.9)	85.2 (80.8–89.6)	75.0 (69.5–80.5)	65.2 (58.7–71.7)	< 0.001
KOOS-ADL (0–100)	76.7 (73.0–80.4)	88.3 (82.2–94.4)	78.5 (73.3–83.7)	65.1 (58.6–71.6)	< 0.001
KOOS-Sport/Rec (0–100)	49.8 (43.9–55.7)	65.4 (53.5–77.3)	52.0 (42.8–61.2)	35.3 (26.7–43.8)	< 0.001
KOOS-QOL (0–100)	60.9 (56.3–65.5)	76.4 (68.4–84.5)	63.0 (56.2–69.7)	46.5 (39.6–53.5)	< 0.001
EQ5D (0–1)	0.75 (0.71–0.79)	0.90 (0.85–0.95)	0.74 (0.69–0.78)	0.63 (0.54–0.72)	< 0.001
SF36-PF (0–100)	72.7 (68.6–76.8)	87.1 (81.8–92.5)	72.1 (66.2–78.0)	62.3 (54.4–70.1)	< 0.001
SF36-RP (0–100)	65.0 (57.7–72.3)	89.8 (80.2–99.4)	61.3 (50.4–72.3)	51.4 (37.2–65.7)	< 0.001
SF36-BP (0–100)	56.2 (51.9–60.5)	83.8 (75.9–91.8)	52.1 (47.3–56.8)	42.1 (36.4–47.8)	< 0.001
SF36-GH (0–100)	66.0 (61.9–70.1)	75.6 (69.2–82.0)	68.9 (63.1–74.7)	53.3 (45.4–61.1)	< 0.001
SF36-VT (0–100)	63.1 (58.6–67.5)	75.0 (67.2–82.7)	64.3 (58.8–70.2)	52.5 (43.6–61.5)	0.002
SF36-SF (0–100)	85.6 (81.7–89.6)	93.5 (87.2–99.9)	87.5 (83.1–91.9)	76.7 (66.9–86.6)	0.018
SF36-RE (0–100)	80.4 (73.8–87.0)	85.9 (73.8–98.0)	84.6 (75.7–93.5)	70.4 (56.2–84.6)	0.232
SF36-MH (0–100)	79.9 (76.7–83.0)	86.5 (80.0–92.9)	81.6 (77.5–85.8)	72.2 (65.9–78.5)	0.001

*NCP* No chronic pain, *CRP* chronic regional pain, *CWP* chronic widespread pain, *BMI* body mass index, *TFOA* radiographic tibiofemoral osteoarthritis, *PFOA* radiographic patellofemoral osteoarthritis, *TFPFOA* radiographic tibio- and patellofemoral osteoarthritis, *OA* osteoarthritis, *TF* tibiofemoral, *PF* patellofemoral, *TFPF* tibio- and patellofemoral, *ADL* activity of daily living, *Sport/rec* function in sport and recreation, *QOL* knee related Quality of life, *PF* physical function, *RP* role-physical, *BP* bodily pain, *GH* general health, *VT* vitality, *SF* social functioning, *RE* role-emotional, *MH* mental health

^a^overall p-value, chi^2^-test

### Pain, radiographic features and BMI

There was no statistically significant difference in the rate of radiographic OA between the groups with NCP, CRP or CWP, neither in the presence of osteophytes or in mean BMI between the groups, Table 1. In total 27% of the participants were obese, though there were no statistically significant difference between the groups (NCP 16%, CRP 31% and CWP 29%). There were no associations between CWP, radiographic OA (OR 1.122, 95% CI 0.468–2.691), osteophytes (OR 0.864, 95% CI 0.198–3.770) or BMI (OR 1.062, 95% CI 0.960–1.174), controlled for age and gender.

### Pain groups and KOOS

**Fig. 1:**
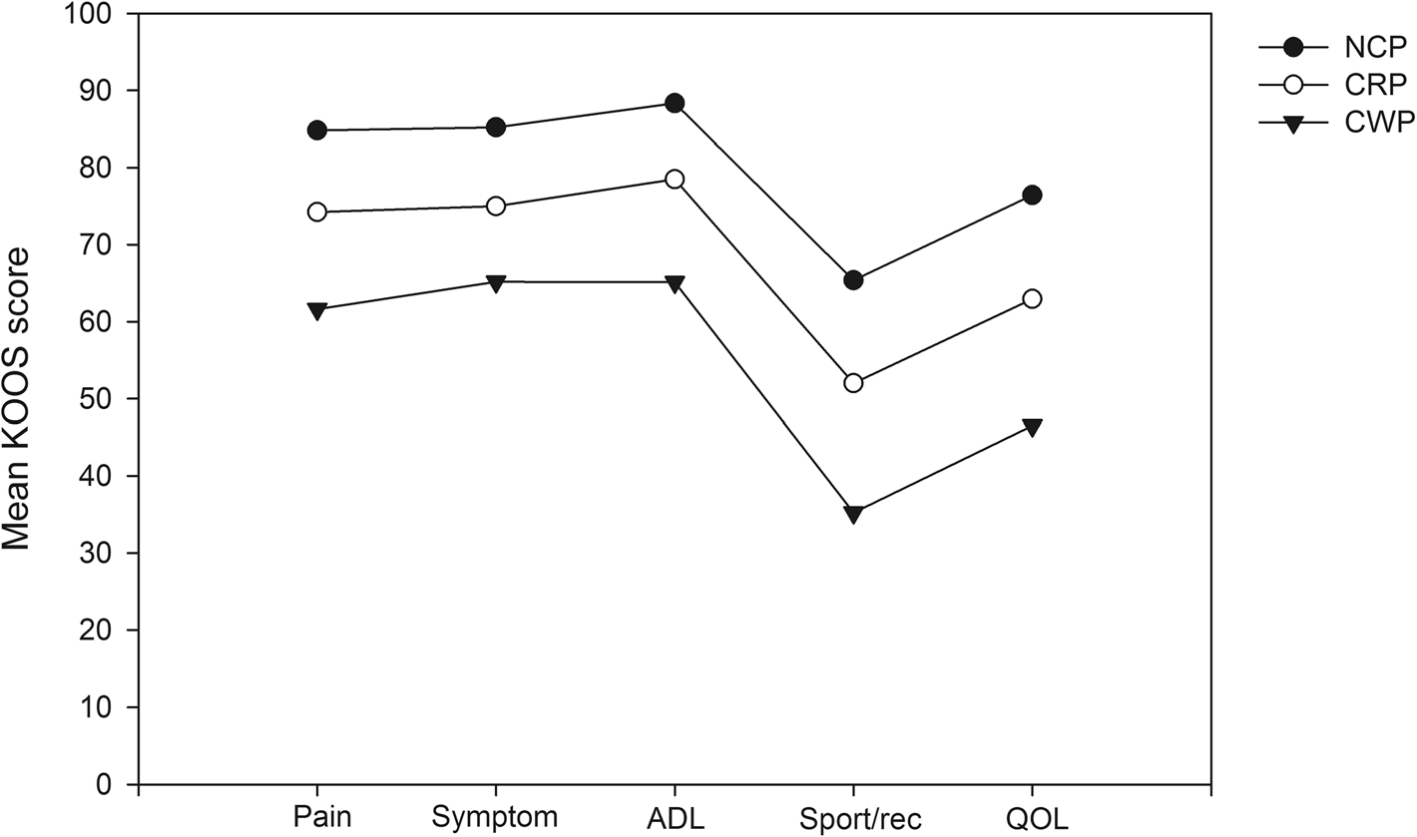
Mean KOOS score in the three groups; NCP, no chronic pain; CRP, chronic regional pain and CWP, chronic widespread pain at 20-year follow-up in individuals with chronic knee pain at inclusion

Individuals reporting CWP had worse KOOS-pain compared to those with NCP and CRP, (*p* < 0.001 and *p* = 0.005), worse KOOS-symptom (*p* = 0.001 and *p* = 0.012), worse KOOS-ADL (p < 0.001 and *p* = 0.002), worse KOOS-sport/rec (p < 0.001 and *P* = 0.008) and worse KOOS-QOL (p < 0.001 and *p* = 0.004), Table 1 and Fig. 1.

In a multiple logistic regression analysis, CWP was associated to being in the worse half of all subgroups of KOOS (pain, symptom, ADL, sport/rec and QOL) controlled for age, gender and radiographic knee OA, Table 2. Having radiographic knee OA was also associated to being in the worse half of all KOOS subscales, Table 2.

**Table 2:** Associations between being in the worse half of KOOS subscales and the different pain groups controlled for age, gender and having or not having radiographic knee OA at 20-year follow-up in a cohort with chronic knee pain at inclusion

	KOOS Pain		KOOS Symptom		KOOS ADL		KOOS Sport/Rec		KOOS QOL	
	OR	95% CI	OR	95% CI	OR	95% CI	OR	95% CI	OR	95% CI
Age	1.003	0.929–1.082	0.974	0.899–1.055	1.015	0.936–1.099	1.021	0.947–1.100	0.993	0.917–1.076
Gender (women)	0.716	0.296–1.733	0.679	0.268–1.724	0.685	0.269–1.749	0.597	0.245–1.453	0.421	0.159–1.117
Knee OA	2.431	1.008–5.865	3.804	1.483–9.761	2.917	1.148–7.414	3.075	1.279–7.396	3.938	1.498–10.353
NCP	1		1		1		1		1	
CRP	2.088	0.686–6.355	3.342	0.943–11.842	2.612	0.809–8.432	1.894	0.627–5.722	3.191	0.880–11.568
CWP	6.379	1.877–21.676	11.958	3.002–47.584	17.295	4.332–69.052	3.522	1.063–11.666	11.300	2.769–46.107

*ADL* activity of daily living, *Sport/Rec* sport and recreation function, *QOL* knee-related quality of life, *CRP* chronic regional pain, *CWP* chronic widespread pain, *Knee OA* having radiographic knee osteoarthritis

### Pain groups and health-related quality of life

Individuals reporting CWP had worse EQ5D compared to those reporting both CRP and CWP (*p* < 0.001 and *p* = 0.027). CWP was associated to being in the worse half of EQ5D (OR 14.6, 95% CI 3.5–61.0), controlled for age, gender and radiographic knee OA.

**Fig. 2:**
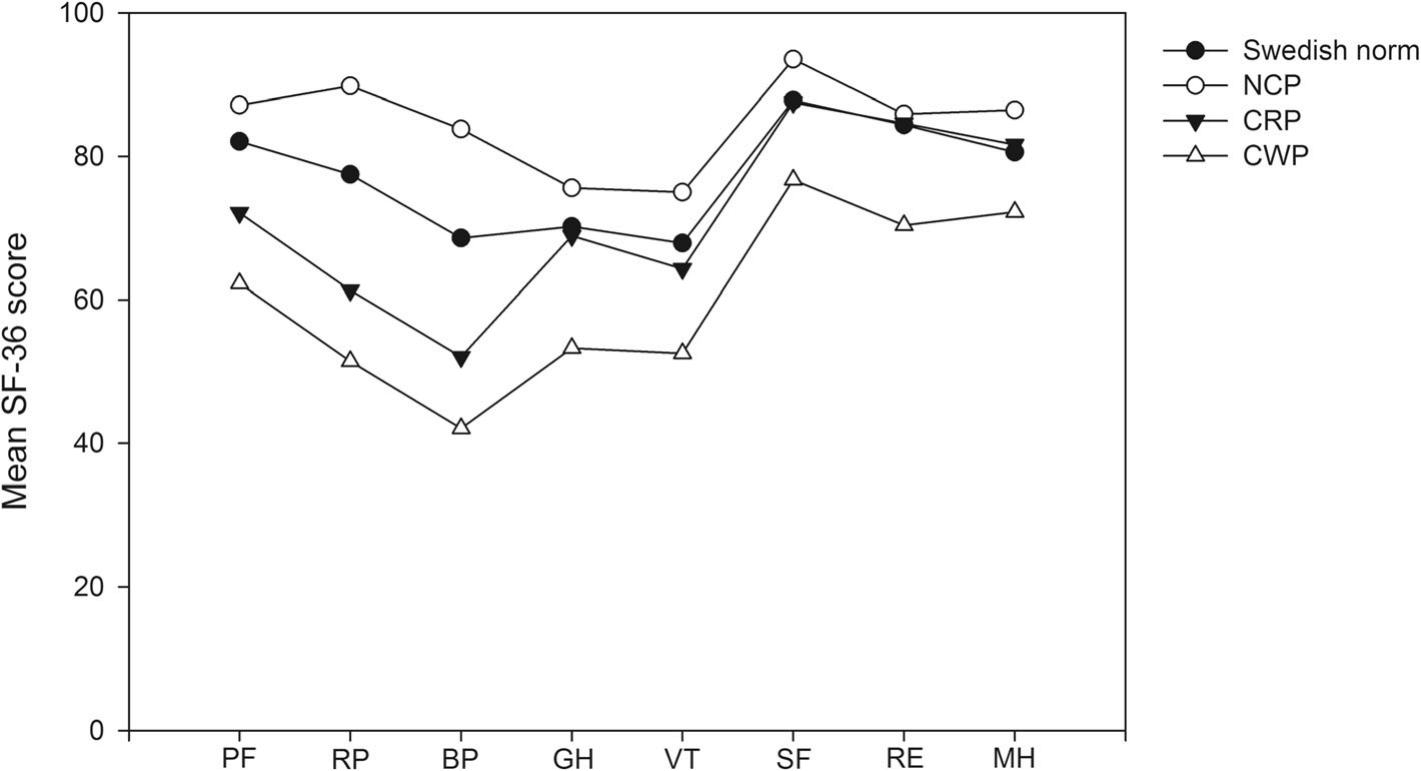
Mean SF-36 score in the three groups; NCP, no chronic pain; CRP, chronic regional pain and CWP, chronic widespread pain at 20-year follow-up of individuals with chronic knee pain at inclusion. Swedish norm from Sullivan et al., 2002 [28]

Individuals reporting CWP had worse SF36 than those reporting NCP in the subscales PF (p < 0.001), RP (p < 0.001), BP (p < 0.001), GH (*p* = 0.001), VT (*p* = 0.002), SF (*p* = 0.014), and MH (p = 0.001), Table 1 and Fig. 2. In the two subscales, GH and MH, individuals reporting CWP also had a worse score than those with CRP (GH *p* = 0.009, MH *p* = 0.035), Table 1 and Fig. 2. There was no significant difference between the groups in the subscale RE.

In multiple logistic regression analyses associations were found between CWP and worse scores in seven of the subgroups of SF-36 (PF, RP, BP, GH, VT, SF, MH), controlled for age, gender and radiographic knee OA, Table 3.

**Table 3:** Associations between being in the worse half of SF-36 subscales and the different pain groups controlled for age, gender and having or not having radiographic knee OA at 20-year follow-up in a cohort with chronic knee pain at inclusion

	SF-36 PF		SF-36 RP		SF-36 BP		SF-36 GH	
	OR	95% CI	OR	95% CI	OR	95% CI	OR	95% CI
Age	1.098	1.014–1.189	1.031	0.952–1.115	0.962	0.886–1.044	1.041	0.962–1.126
Gender (Women)	1.542	0.618–3.845	0.840	0.339–2.084	1.238	0.476–3.220	0.688	0.275–1.723
Knee OA	1.536	0.627–3.764	1.420	0.581–3.471	0.883	0.344–2.264	1.064	0.434–2.613
NCP	1		1		1		1	
CRP	4.080	1.248–13.342	7.736	2.153–25.269	20.056	4.071–98.795	2.370	0.792–6.234
CWP	9.391	2.491–35.400	11.654	2.854–41.544	45.482	6.413–185.387	13.348	3.467–51.396
	**SF-36 VT**		**SF-36 SF**		**SF-36 RE**		**SF-36 MH**	
	OR	95% CI	OR	95% CI	OR	95% CI	OR	95% CI
Age	1.005	0.933–1.082	1.014	0.943–1.091	0.996	0.918–1.081	**1.095**	**1.008–1.190**
Gender (women)	1.208	0.513–2.844	0.750	0.320–1.759	1.223	0.483–3.095	2.140	0.837–5.470
Knee OA	0.887	0.378–2.081	1.049	0.452–2.438	1.540	0.597–3.971	1.284	0.512–3.217
NCP	1		1		1		1	
CRP	3.707	1.171–11.741	2.608	0.831–8.190	1.089	0.322–3.681	4.260	1.292–14.044
CWP	6.560	1.897–22.686	4.036	1.196–13.612	2.235	0.648–7.705	12.238	3.131–47.830

*PF* physical function, *RP* role physical, *BP* bodily pain, *GH* general health, *VT* vitality, *SF* social functioning, *RE* role emotional, *MH* mental health, *CRP* chronic regional pain, *CWP* chronic widespread pain, *Knee OA* having radiographic knee osteoarthritis

## Discussion

In this cross-sectional study of individuals with knee pain at inclusion, one third reported CWP at a 20-year follow-up, regardless of having radiographic knee OA or not. The presence of CWP was associated to worse outcome in KOOS, SF36 and EQ5D.

There are no comparable studies on OA and CWP, but studies in the general population have reported a prevalence of CWP between 11 and 13% [3, 33, 34], with an overall prevalence in the world of 10% [6]. In RA the prevalence of CWP has been reported to be in the same order as for OA in this study, about 30% [15].

In the present study 6% reported that they had fibromyalgia, which is somewhat lower than previously reported in patients with OA [2], but higher than the prevalence in the general population, reported to be between 1 to 5% [3–5]. The difference could depend on that the diagnose was self-reported in the present study and not evaluated by clinical examination [2]. The prevalence of fibromyalgia is also increased compared to general population in other rheumatic diseases. For example, in SLE and AS about 13% are fulfilling the criteria for fibromyalgia and in Sjögrens syndrome about 12% [2].

In this study, no associations between CWP and radiographic changes were found, neither when assessed by joint space width or by osteophytes. There have been divergent results when studying the association between pain, radiographic OA and osteophytes, [35–38]. Though, a study by Finan et al. reported that central sensitization was more frequently present in patients, who reported a high level of clinical pain in the absence of moderate-to-severe radiographic knee OA [7].

In the present study there was no difference in BMI between the three pain groups, although there was a numerically higher rate of obesity in the CRP and CWP groups than in the NCP group. A study from the Osteoarthritis Initiative has shown that individuals with higher BMIs reported more pain than individuals with lower BMIs [39].

Individuals reporting CWP had worse KOOS in all subscales compared to those not reporting any chronic widespread pain. In KOOS, all subscales were associated to both knee OA and chronic widespread pain, with the highest association between CWP and activity of daily living. Another study has shown similar finding in individuals with OA and fibromyalgia reporting worse WOMAC compared to individuals with OA without fibromyalgia [2]. Individuals reporting NCP had better health-related quality of life than both CRP and CWP and that is in line with results from other studies [40, 41]. Pain is probably one of the most important factors affecting function, well-being and health-related quality of life regardless of the chronic disease [42–44]. The association between patient reported outcome in rheumatic disorders and chronic widespread pain is well known. Disease specific measurements, such as 28-joints Disease Activity Score (DAS28) for rheumatoid arthritis (RA) and Bath Ankylosing Spondylitis Disease Activity Index (BASDAI) for ankylosing spondylitis (AS), have also been shown to be influenced by fibromyalgia or CWP, were patients with fibromyalgia or CWP reported worse disease activity [2, 15, 17]. Pain affects patients’ perception of function and well-being significantly. In both clinic and research, when using knee specific assessments as KOOS and WOMAC, the interpretation is that it is the knee problems that is measured, although it is common to have pain elsewhere as well. The minimum clinically important difference (MCID) reported for KOOS is ≥20 [45]. The difference between the NCP and the CWP group in all subscales are ≥20. The minimal important change (MIC) is suggested to be 8–10 in all subscales [46]. The differences between the groups in this study are above MIC in all subscales. Since there are differences between groups that are above MIC and in some cases above MCID, CWP could be considered to affect KOOS in a clinically relevant way. A clinical implication of this could be that even if you treat the knee symptoms, it may not have a substantial effect on the proposed knee related scores due to that pain in other sites also has an impact on the score. When using these instruments for assessing disease activity both in clinical practise and in research it is important to assess and be aware of that generalized pain is a common coexisting phenomenon. One way to investigate if the patient has pain in other sites could be to ask the patient in a structured way and document it or to ask the patient to fill in a pain mannequin and take the results in to account when evaluating the results from KOOS. From a clinical point of view it is also important to identify individuals with knee pain and a concomitant CWP, since they may need a more complex intervention [47].

[40–44]The cross-sectional design is a limitation that precludes assessment of causal relationships. The study is based on data collected in 2010, which could be considered to be a limitation due to time. However, the association between knee pain and CWP could still be considered to be important and relevant when assessing knee pain in the clinic.

## Conclusion

One third of individuals with chronic knee pain met the criteria for CWP. CWP was associated with patient reported pain, function and health-related quality of life. This suggest that it is important to assess CWP in the evaluation of patients with chronic knee pain with and without radiographic knee OA, when evaluating knee related outcomes in research and clinical settings.

## Abbreviations

ADL: Function in daily living
AS: Ankylosing spondylitis
BASDI: Bath Ankylosing Spondylitis Disease Activity Index
BMI: Body mass index
BP: Bodily pain
CRP: Chronic regional pain
CWP: Chronic widespread pain
DAS28: 28-joints Disease activity index
EQ-5D: Euroqol 5D-3 L
FM: Fibromyalgia
GH: General health
HRQL: Health related quality of life
JSW: Joint space width
KOOS: Knee injury and Osteoarthritis Outcome Score
MH: Mental health
NCP: No chronic pain
OA: Osteoarthritis
PF: Physical functioning
PFOA: Patellofemoral osteoarthritis
QOL: Knee related Quality of life
RA: Rheumatoid arthritis
RE: Role emotional
RP: Role physical
SF: Social functioning
SF36: Short form 36
Sport/rec: Function in sport and recreation
TFOA: Tibiofemoral osteoarthritis
VT: Vitality
